# Supplementary material for: Degron masking outlines degronons, co-degrading functional modules in the proteome
Source: Commun Biol. 2022 May 11;5:445. doi: 10.1038/s42003-022-03391-z (PMC9095673; doi:10.1038/s42003-022-03391-z)
Supplement: Supplementary file 2 — Supplementary Information [file 42003_2022_3391_MOESM2_ESM.pdf]

Supplementary Information

Degron masking outlines degronons, co-degrading functional modules in the proteome

Mainak Guharoy[1,2,3,*], Tamas Lazar[1,2], Mauricio Macossay-Castillo[1,2] and Peter Tompa[1,2,4,*]

1 VIB-VUB Center for Structural Biology, Pleinlaan 2, 1050 Brussels, Belgium.

2 Structural Biology Brussels, Department of Bioengineering Sciences, Vrije Universiteit Brussel, Pleinlaan 2, 1050 Brussels, Belgium.

3 VIB Bioinformatics Core, Technologiepark-Zwijnaarde 75, 9052 Ghent, Belgium.

4 Institute of Enzymology, Research Centre for Natural Sciences of the Hungarian Academy of Sciences, 1117 Budapest, Hungary.

*Correspondence: mainak.guharoy@vib.be (MG), peter.tompa@vub.be (PT)

Contents

Supplementary Figure 1	page 3
Supplementary Figure 2	page 4
Supplementary Figure 3	page 5
Supplementary Figure 4	page 6
Supplementary Figure 5	page 7
Supplementary Figure 6	page 8
Supplementary Figure 7	page 9
Supplementary Figure 8	page 10
Supplementary Figure 9	page 11
Supplementary Figure 10	page 12
Supplementary Figure 11	page 13
Supplementary Figure 12	page 14
Supplementary Figure 13	page 15
Supplementary Table 1	pages 16-18
Supplementary Discussion	page 19
Supplementary References	page 20-25.

Supplementary Figure 1. The Mdm2-binding primary degron of p53 (**a**) (in yellow, amino acids 19-26) is masked in multiple complexes formed with different domains of CREB-binding protein, CBP. (**b-d**) NMR ensembles of the disordered N-terminal transactivation (TAD) domain of P53 (in red, containing the degron) in complex with multiple partner domains (in grey) of CBP. Two views are shown for each complex: both partners in cartoon representation (left) and the degron masking domains in surface representation (right). PDB ids of each complex are provided within brackets.

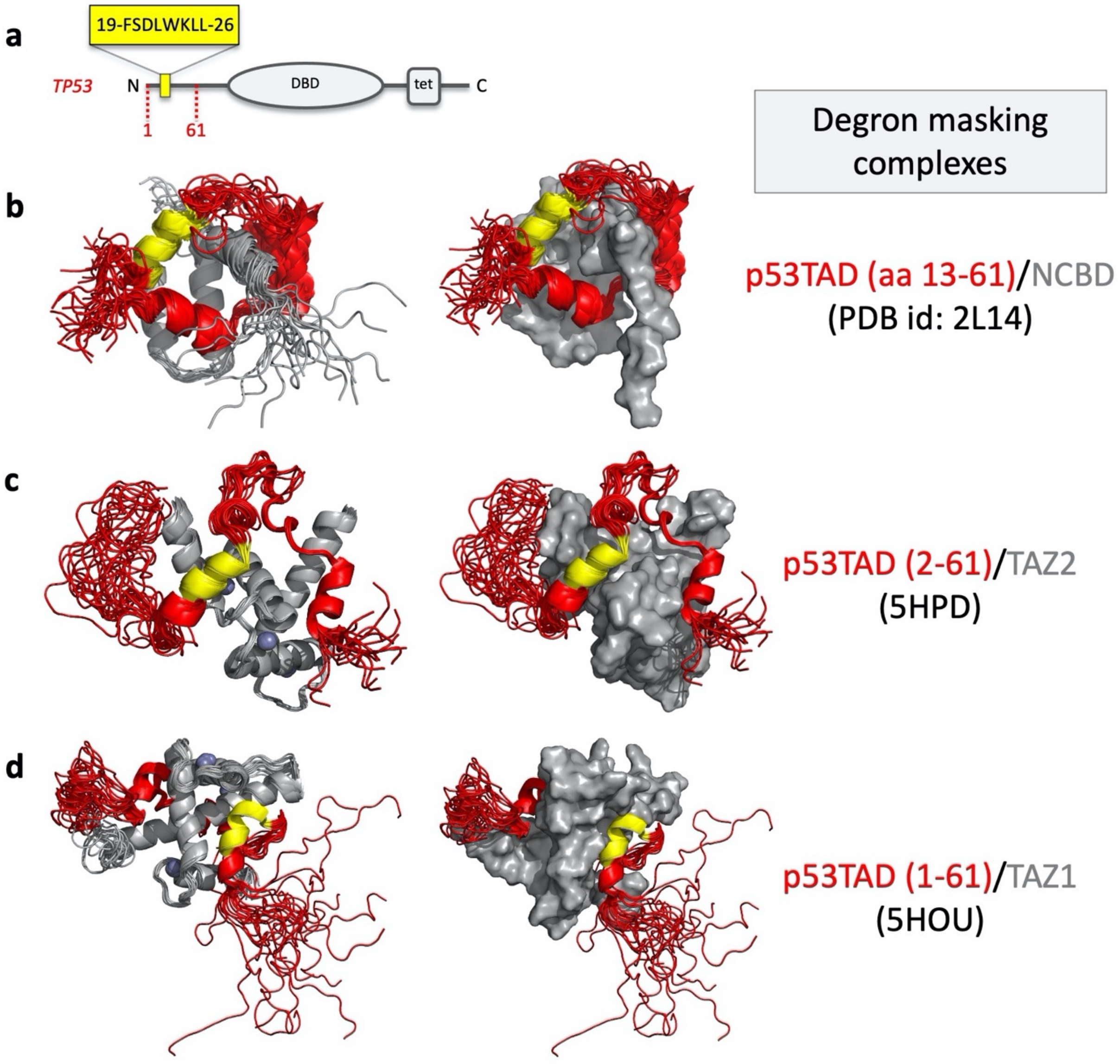

Supplementary Figure 2. IUPred[1] disorder profile (**a**) for the YAP1 protein. The secondary degron is K97 which is located within the IDR (amino acids 80-171, region is shaded red). This IDR is also predicted to be the tertiary degron of YAP1. Domain diagram of YAP1 (**b**) and (**c**) IntAct[2] annotated binding sites of TEAD2 and TEAD4. The binding sites of both proteins mask the secondary as well as tertiary degrons of YAP1.

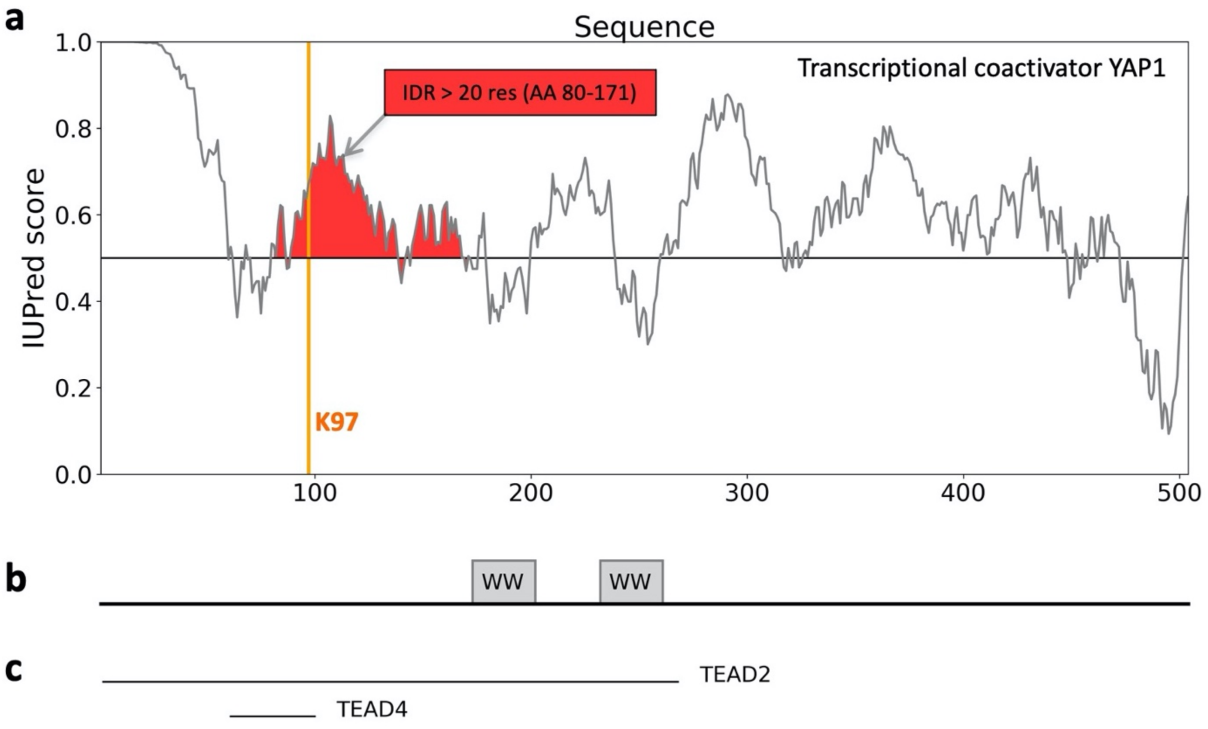

Supplementary Figure 3. Pairwise functional similarity between substrate proteins and their interacting partner proteins (*left* boxes) and substrate-degron masking partners (*right* boxes). The subplots from left to right correspond to substrates with annotated primary, secondary and tertiary degrons, respectively. The proteins correspond to those analyzed in **Figure 2**. Functional similarity of each interacting protein pair (plotted along the y-axis) was calculated using pairwise Gene Ontology (GO) Biological Process semantic similarity scores; details are provided in the Methods section. A higher score indicates greater functional similarity between the two proteins. Below each box is shown the number of protein pairs corresponding to that category. The Mann-Whitney U-test p-values, measuring the statistical significance of the differences between the two distributions, are shown above each plot.

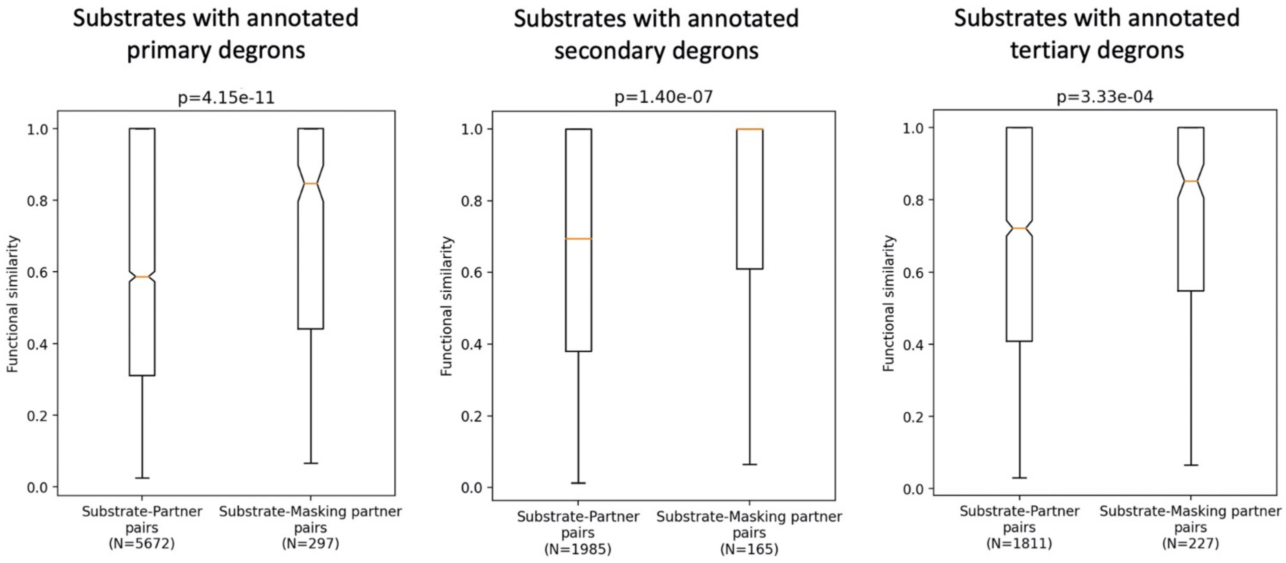

Supplementary Figure 4. IUPred[1] disorder profile (**a**) for the beta-catenin protein. All three degron components are located proximally in this protein. Primary degron (in yellow, amino acids 32-37), the secondary degrons are K19 and K49 and the predicted tertiary degron is the IDR nearest to these lysines, i.e., amino acids 32-64 (region shaded red). Domain diagram of beta-catenin (**b**) and (**c**) IntAct[2] annotated binding sites demonstrate that the binding sites of multiple partners overlap with all three tripartite degron elements.

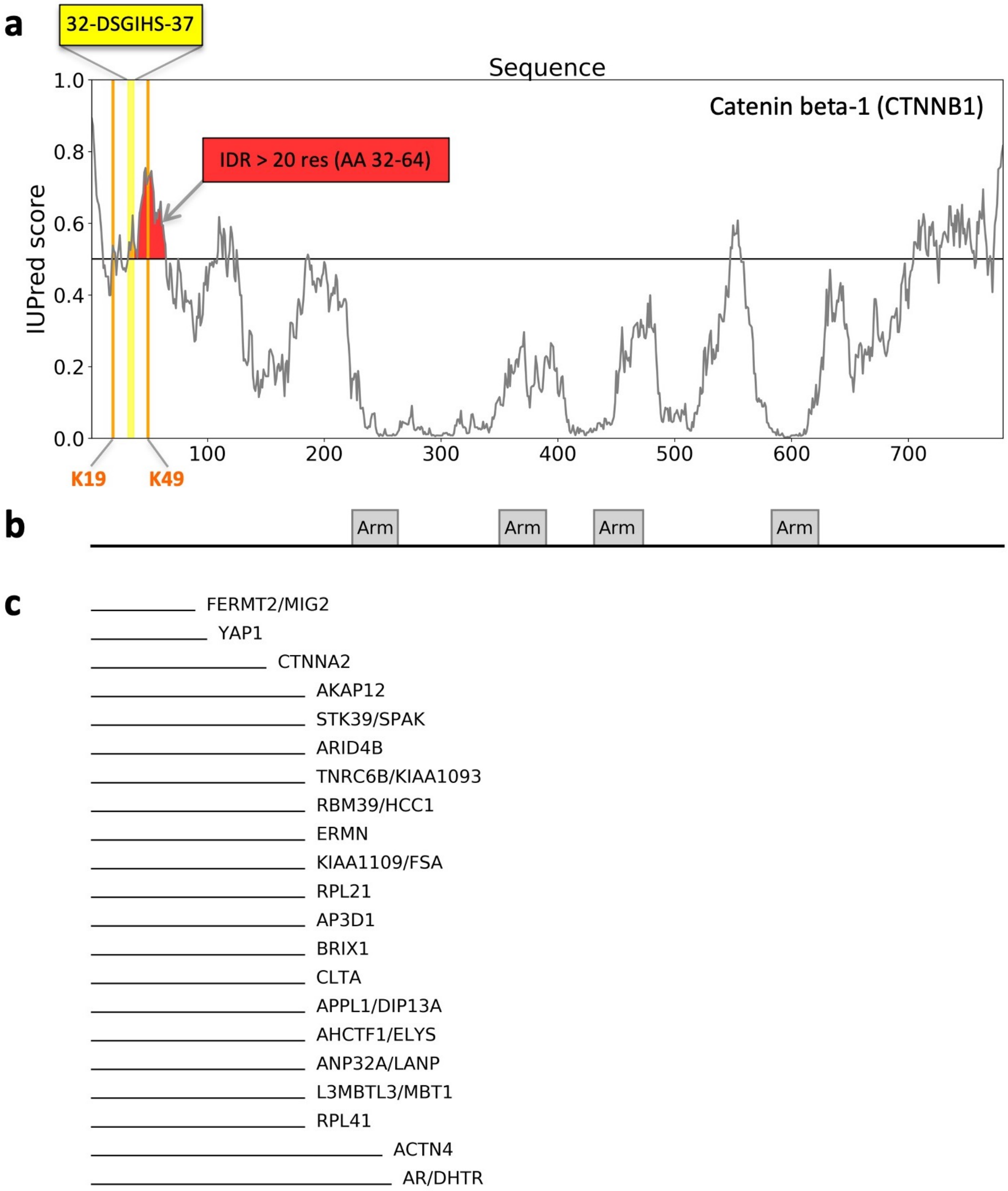

Supplementary Figure 5. IUPred[1] disorder profile (**a**) for the Hif1a protein. All three degron components are located proximally in this protein. Two primary degrons (in yellow, amino acids 400-413 and 562-574), the secondary degrons K532,538 and 547 and the predicted tertiary degron is the IDR nearest to these lysines, i.e., amino acids 442-521 (region shaded red). Domain diagram of Hif1a (**b**) and (**c**) IntAct[2] annotated binding site data showing that the binding sites of several partners overlap multiple degrons simultaneously.

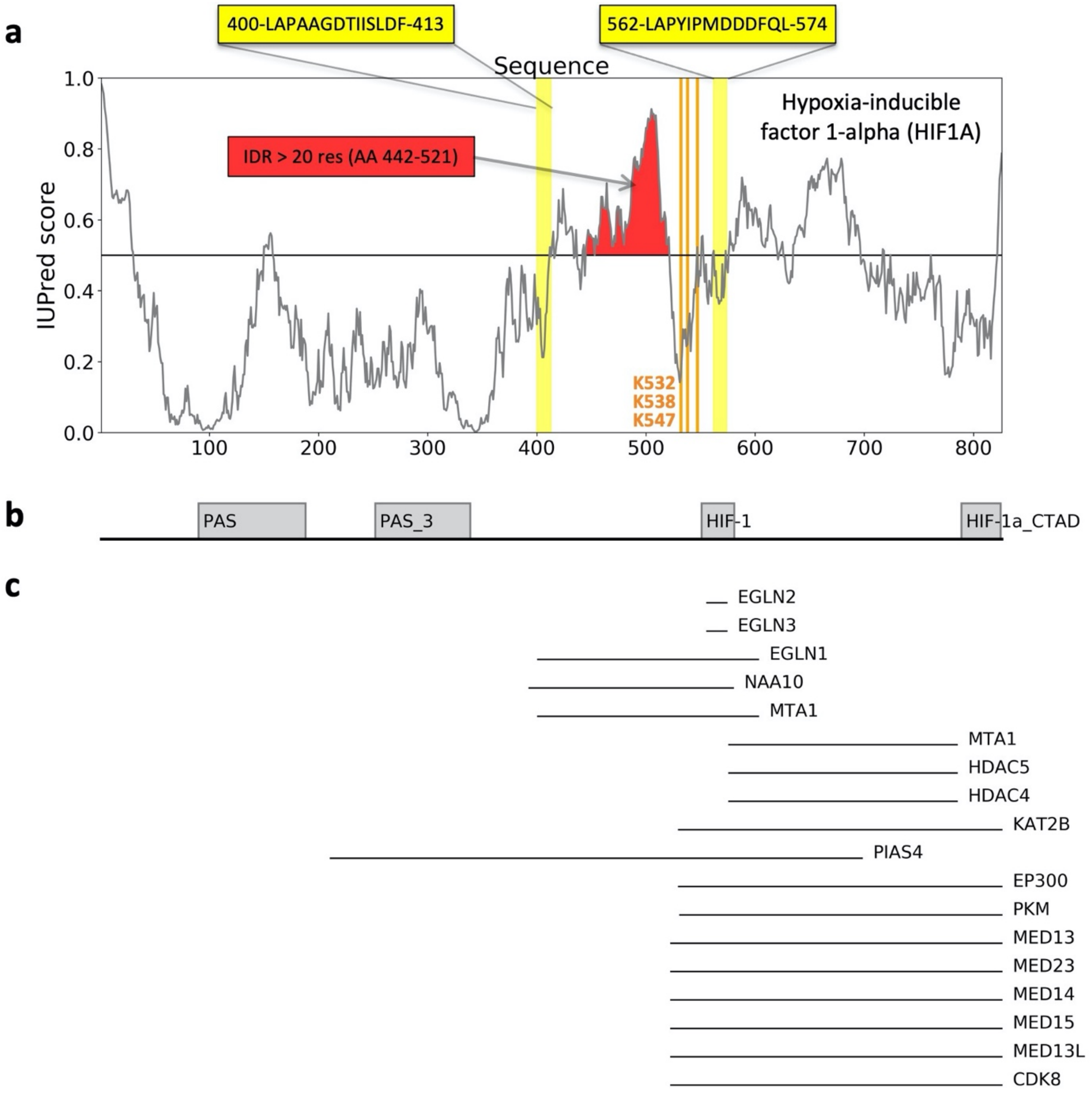

Supplementary Figure 6. IntAct[2] binding data details. (**a**) Occurrences (counts) of the various binding site related terms as annotated by IntAct, (**b**) length distribution of binding sites that overlap with degrons, and (**c**) distribution of IntAct interaction confidence scores (we chose a cutoff of 0.3).

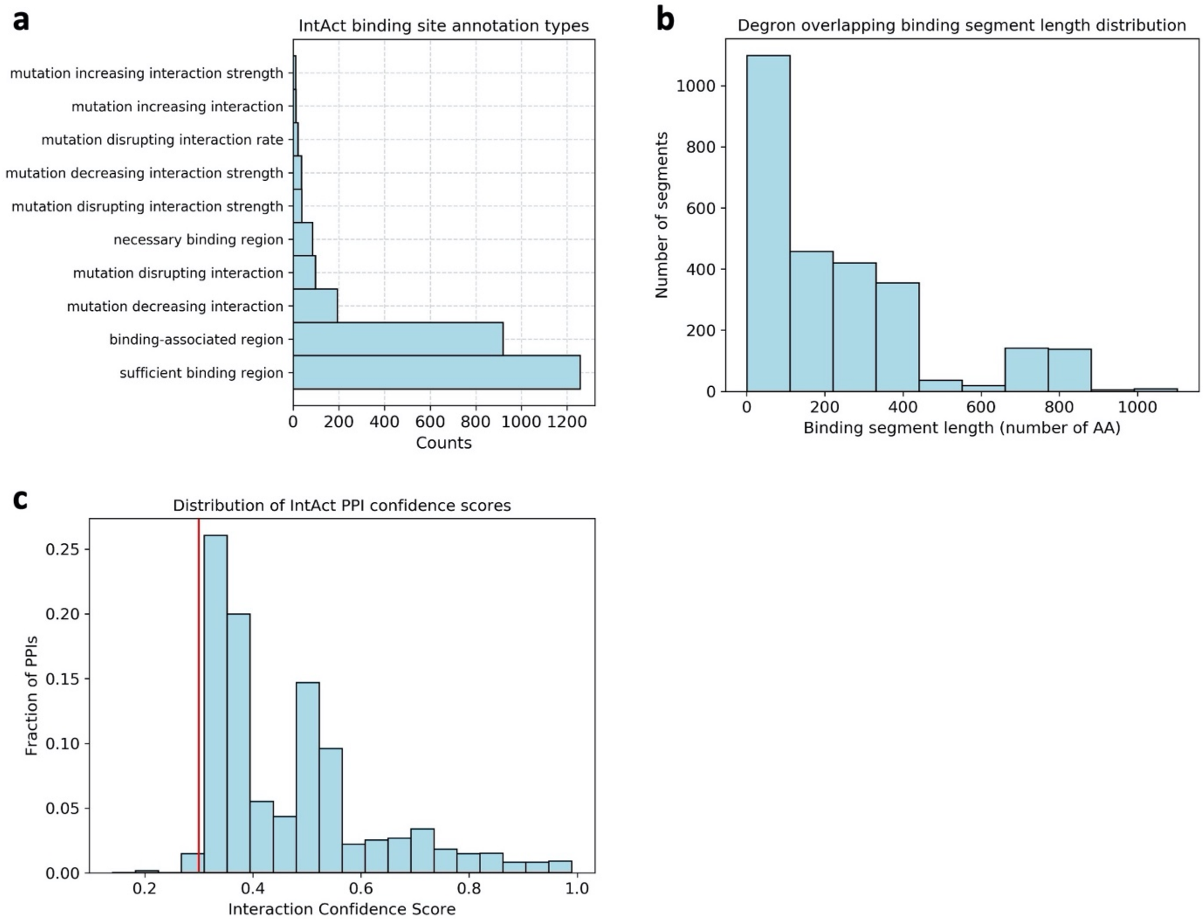

Supplementary Figure 7. Comparison of dissociation constants (Kd) values for binding of substrates with their cognate E3 ligases and between substrates and their degron-masking alternative partners (APs). The details of the Kd measurements are provided in **Supplementary Data 9**. (**a**) Boxplot showing the distributions of Kd values for substrate-E3 (*left*) and substrate-AP (*right*) interactions. If more than one measurement was available for a given interaction (**Supplementary Data 9**), the mean Kd value was calculated and plotted. The difference between the two distributions is not statistically significant (P=0.23). (**b**) Scatter plot of six systems where binding affinities of both E3 ligases and APs are known for the same substrates. Substrate names are shown in black, E3s in red and AP name(s) in blue. When multiple Kd values were available for the same interaction or for interactions of one substrate with multiple APs (**Supplementary Data 9**), the mean Kd value was plotted, with the standard error shown as whiskers. The abundances (in parts per million, ppm) of each denoted protein is shown next to its name. Abundance values were obtained from the PaxDb database[3], from the human whole organism integrated dataset (see Methods section); E3 ligases and their abundances shown in red, APs in blue.

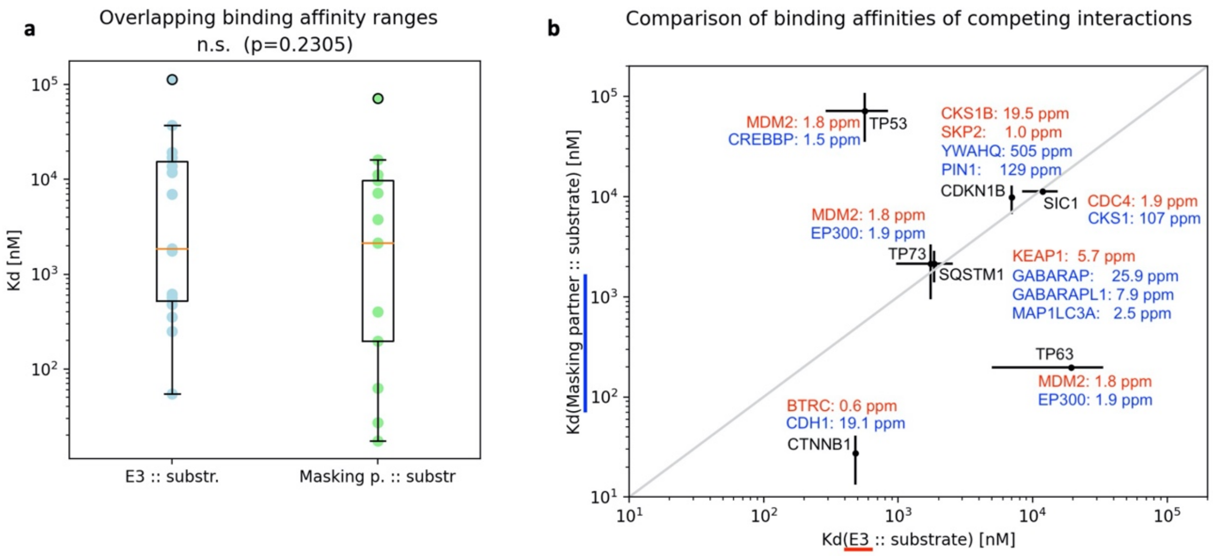

Supplementary Figure 8. PaxDb[3] (ppm) abundances of substrates, E3 ligases and alternative (degron masking) partners, APs (from **Table 1**). The abundance distributions are shown per dataset group (**a**) for whole organism and integrated datasets, and (**b**) tissue and cell line datasets. The whole proteome abundance distribution is shown for comparison purposes. Abundance dataset details are provided in **Supplementary Data 10**.

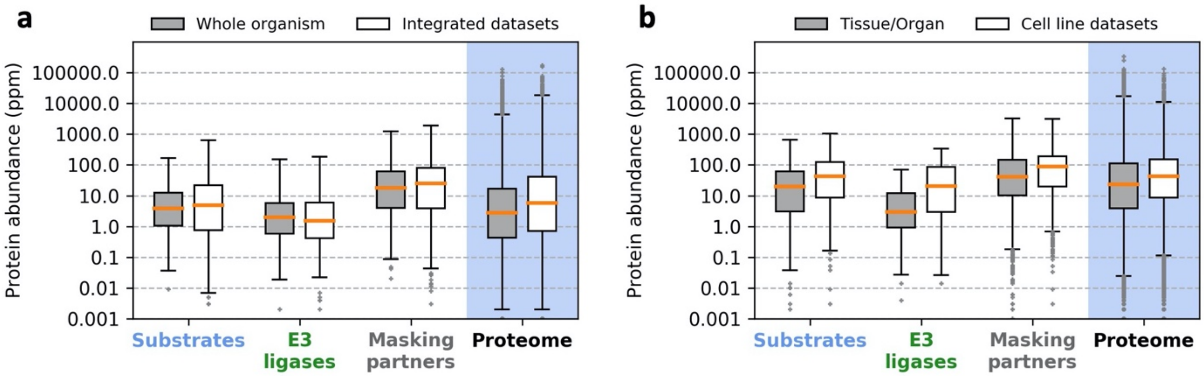

Supplementary Figure 9. Distributions of ranked (binned) abundances of substrates, E3 ligases and alternative (degron masking) partners, APs (from **Table 1**). The conversion of ppm abundances (provided by PaxDb[3]) into ranked abundances is described in Methods (section 'Protein abundance data'). The ranked abundance distributions are shown per dataset group (**a**) whole organism and integrated datasets, and (**b**) tissue and cell line datasets. Abundance dataset details are provided in **Supplementary Data 10**.

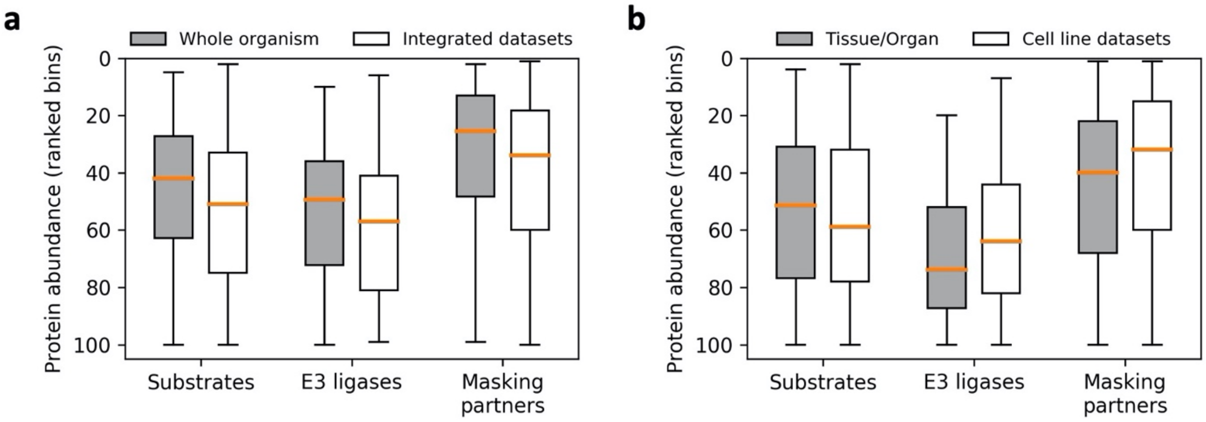

Supplementary Figure 10. Scatter plot of substrate-AP paired abundances (across all 170 PaxDb[3] datasets). Abundances were taken from each individual PaxDb dataset, whenever data for both proteins of a pair (i.e., substrate, AP) were available. Spearman's correlation coefficients (rS) and corresponding p-value are shown.

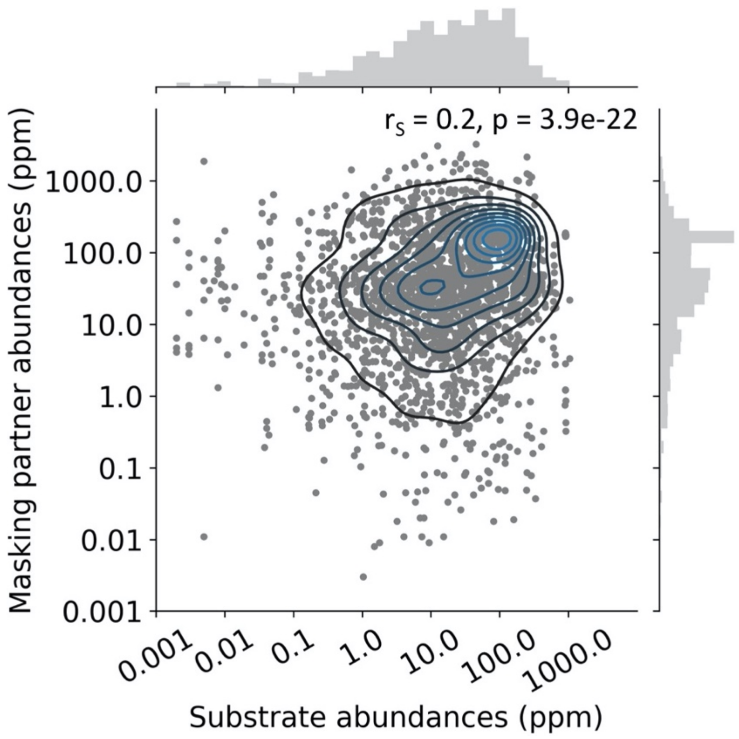

Supplementary Figure 11. Abundance variations of (**a**) E3s and (**b**) degron-masking partners (APs) (listed in **Table 1**) across tissue (dark grey) and cell line (light grey) PaxDb[3] datasets.

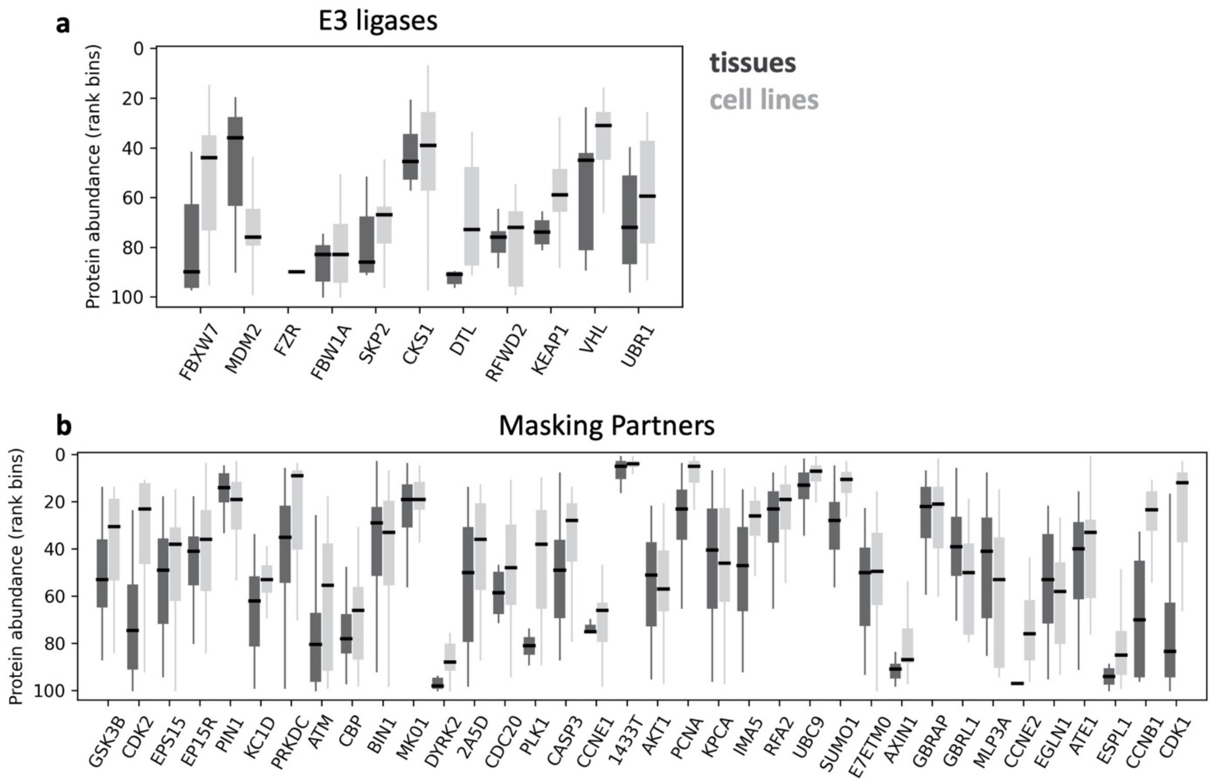

Supplementary Figure 12. Distribution of half-life ($T_{1/2}$) ratios of PPI pairs compared versus those of PPI pairs from randomized networks (average ±1 standard deviation range, calculated on 10 random networks, shown in red). The PPI network and $T_{1/2}$ datasets used were as follows (also mentioned on the figure panels): (**a**) PPI network (Collins), $T_{1/2}$ dataset (Christiano), (**b**) PPI network (BioGRID), $T_{1/2}$ dataset (Martin-Perez) and (**c**) PPI network (BioGRID), $T_{1/2}$ dataset (Christiano). The details of the PPI networks and the $T_{1/2}$ datasets are described in the Methods section.

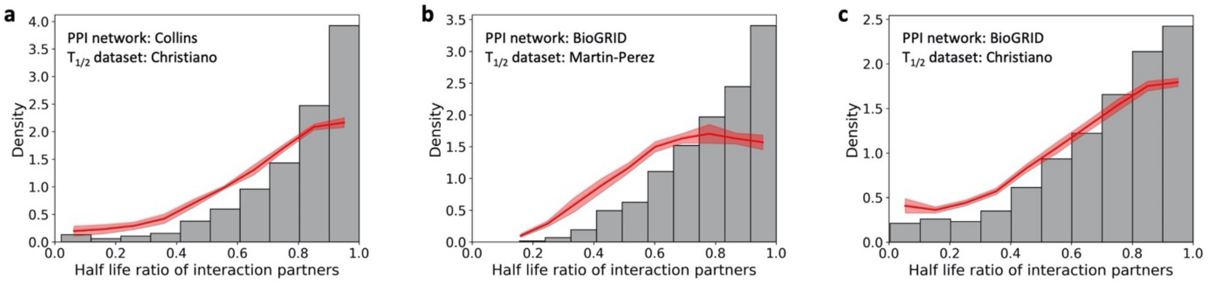

Supplementary Figure 13. PaxDb[3] dataset statistics: (**a**) dataset interaction consistency scores, (**b**) summed abundances of all proteins quantified, calculated per dataset, (**c**) PaxDb dataset coverage, and (**d**) dataset size (number of proteins present).

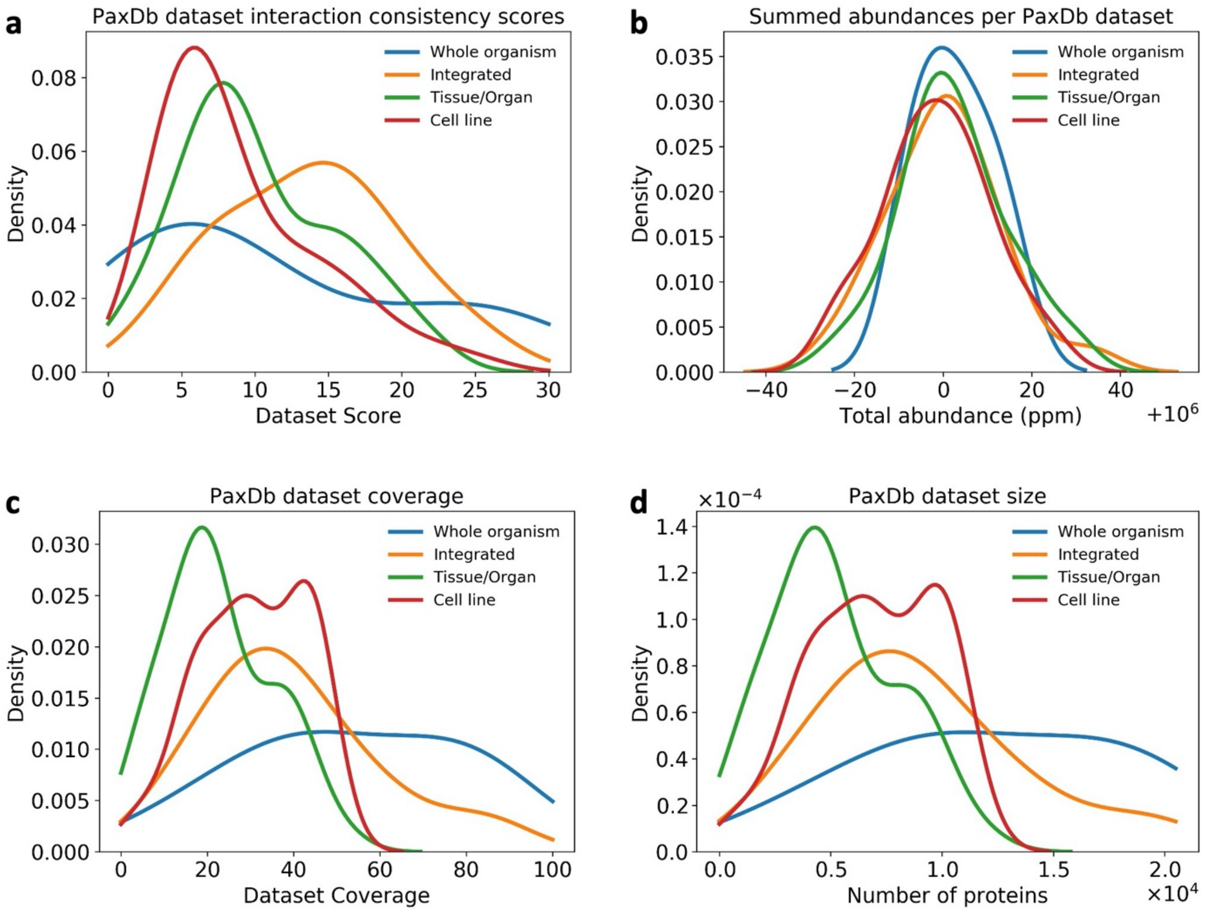

Supplementary Table 1. Literature curated list of proteins stabilized by interactions with specific partner proteins.

Degradation Substrate	Stabilizing Partner	References
Matα2	Mata1	Johnson et al.[4]
Matα2	Tup1	Laney et al.[5]
Matα2	Mcm1	Hickey & Hochstrasser[6]
Matα2	Ssn6	Hickey & Hochstrasser[6]
AURKA	TPX2	Giubettini et al.[7]
DLGAP5/HURP	KPNB1	Song & Rape[8]
NUSAP1	KPNB1	Song & Rape[8]
ZC3HC1/hNIPA	SKP1	Klitzing et al.[9]
BRCA1	BARD1	Wu et al.[10]
α3 nAChR subunit	UBXD4	Rezvani et al.[11]
$GABA_A$ receptor	Plic-1	Bedford et al.[12], Saliba et al.[13]
NMDA receptor subunit NR1	Neurofilament-light (NF-L)	Ratnam & Teichberg[14]
GLUT4	TUG	Yu et al.[15]
mGluR1α receptor	Homer-1A	Soloviev[16]
T cell receptor complex α subunit	CD3-δ subunit	Bonifacino et al.[17]
Ndc10	components of the centromere DNA-binding protein complex, CBF3	Kopski & Huffaker[18]
Elk-1	Dimerization	Evans et al.[19]
C/EBPdelta	Dimerization	Zhou & Dewille[20]
ETS2	Mutant p53	Carrero et al.[21]
Paip2	PABP	Yoshida et al.[22]
E2F1	pRB	Campanero & Flemington[23], Hofmann et al.[24]
E2F4	p107	Hateboer et al.[25]
E2F4	p130	Hateboer et al.[25]
EID1	pRB	Miyake et al.[26]
EID1	Necdin	Bush & Wevrick[27], Zhang et al.[28]
IκBα	NF-κB	Mathes et al.[29], Fortmann et al.[30]
β-catenin	Nek2	Mbom et al.[31]
Cog1	Cog2, Cog3	Shemorry et al.[32]
Hcn1	Cut9	Shemorry et al.[32]
BAF57	BAF155	Keppler & Archer[33]
SNF5	SRG3	Sohn et al.[34]
BRG1	SRG3	Sohn et al.[34]
BAF60a	SRG3	Sohn et al.[34]
LMO2	SCL	Lécuyer et al.[35]

LMO2	SSBP2	Xu et al.[36]
LMO2	Ldb1/CLIM2	Ostendorff et al.[37]
LIM homeodomain (LIM-HD) proteins	CLIM	Güngör et al.[38]
CLIM	single-stranded DNA-binding protein 1 (SSDP1)	Güngör et al.[38]
Ldb1	SSBP2	Xu et al.[36]
Ldb1	LMO2	Hiratani et al.[39]
Apterous	Chip	Weihe et al.[40]
p53	$TAF_{II}31$	Buschmann et al.[41]
p53	Wilms' tumor-suppressor protein WT-1	Maheswaran et al.[42]
p53	HIF-1α	An et al.[43]
p53	p300	Yuan et al.[44]
Mdm2	p300	Kawai et al.[45]
APP-BP1	Uba3	Park et al.[46]
$p19^{ARF}$	Nucleophosmin	Kuo et al.[47]
Homothorax	Extradenticle	Abu-Shaar & Mann[48]
N-Myc	Aurora A	Otto et al.[49]
p63	Cables1	Wang et al.[50]
ribosomal protein S3	Hsp90	Kim et al.[51]
ribosomal protein S6	Hsp90	Kim et al.[51]
human T-cell leukemia virus type 1 (HTLV-1) tax oncoprotein	Hsp90	Gao & Harhaj[52]
HIV-1 integrase	host protein Ku70	Zheng et al.[53]
HIV-1 integrase	LEDGF/p75	Llano et al.[54]
Translationally controlled tumor protein (TCTP)	Hsp27	Baylot et al.[55]
p53	NQO1	Asher et al.[56]
p73α	NQO1	Asher et al.[56]
HIF-1α	MSF-A	Amir et al.[57]
ErbB2	Septin-2	Marcus et al.[58]
BCL2L12, BCL2L12A	Hsp70	Yang et al.[59]
Sit1	Aft1	Kang et al.[60]
Shaker potassium channel protein	Calnexin	Khanna et al.[61]
CCM2	CCM3	Draheim et al.[62]
β-catenin	transducin beta-like 1 (TBL1)	Dimitrova et al.[63]
GPS2	TBL1	Huang et al.[64]
Pen-2	presenilin 1 (PS1)	Crystal et al.[65]
ornithine decarboxylase (ODC), monomeric	NAD(P)H quinone oxidoreductase 1 (NQO1)	Kahana et al.[66]
DNA methyltransferase 1 (DNMT1)-associated protein (DMAP1)	Daxx	Muromoto et al.[67]

P-Glycoprotein (Pgp)	Pim-1	Xie et al.[68]
hepatitis C virus nonstructural protein NS5A	Y-box binding protein 1 (YB-1)	Wang et al.[69]
RIP1	Hsp90	Fearns et al.[70], Lewis et al.[71]
WASF3	Hsp70	Teng et al.[72]
N-WASP	Hsp90	Park et al.[73]
PTEN	homodimerized p85α	Cheung et al.[74]
ELL2	AFF4	Liu et al.[75]
MDM2	CARP1 and CARP2	Yang et al.[76]
Insig-1	Scap/SREBP	Gong et al.[77]
c-IAP1	TNF receptor-associated factor (TRAF) 2	Csomos et al.[78]
ΔNp63α	Yes-associated protein (YAP)	Tomlinson et al.[79]
p73	Yap1	Levy et al.[80]
DNA polymerase β	XRCC1	Fang et al.[81]
XRCC1	Hsp90	Fang et al.[81]
FANCA	Hsp90	Oda et al.[82]
$p22^{phox}$	$gp91^{phox}$	Ellison et al.[83]
Skp2	Cks1	Wang et al.[84]
Tipin	Timeless	Chou & Elledge[85]
Axin	Gsk3	Ji et al.[86]

Supplementary Discussion.

Several substrates (e.g., Bub1, Claspin, Myc, p53) showed significant differences in relative abundances when compared between tissues and cell lines. For example, the expression of the mitotic spindle checkpoint kinase, Bub1 is significantly higher in cell lines relative to normal tissues (**Fig. 5a**). High Bub1 levels, found in many human cancers, impairs chromosomal segregation[87]. However, in certain cases, low Bub1 levels also drive tumorigenesis[88]. Bub1 cell line abundances mirror this: i.e., although, overall Bub1 has higher expression in cancer cell lines, it also covers the lower end of the scale (**Fig. 5a**). Another example is Claspin (CLSPN), a mediator of the replication checkpoint, that is upregulated in many cancers[89,90], and the data reflects a significant trend towards higher abundances in cell lines (**Fig. 5a**). Similarly, Myc is elevated in the majority of cell lines (**Fig. 5a**), matching its known upregulation in cancer[91]. In the case of p53, normal (unstressed) cells have very low p53 levels, due to rapid turnover of the wild-type protein. However, p53 levels in tumor cells are relatively high (**Fig. 5a**), being frequently mutated in cancer, and with many overexpressed p53 mutants exerting oncogenicity[92,93].

The data for E3s and APs also highlights the fact that: (1) the abundances of individual proteins vary considerably within tissue types and cell lines, and (2) their relative abundances often differ significantly when tissues and cell lines are compared (**Supplementary Fig. 11**). Examples include known oncogenes such as the E3 ligases Skp2 (e.g., overexpressed in breast[94] and non-small cell lung cancers[95]), DTL (in hepatocellular[96], breast[97] and gastric[98] carcinomas), and, β-TrCP/Fbw1A[99].

Supplementary References

1. Dosztányi, Z., Csizmok, V., Tompa, P. & Simon, I. IUPred: Web server for the prediction of intrinsically unstructured regions of proteins based on estimated energy content. *Bioinformatics* **21**, 3433–3434 (2005).
2. Orchard, S. *et al.* The MIntAct project - IntAct as a common curation platform for 11 molecular interaction databases. *Nucleic Acids Res.* **42**, D358–D363 (2014).
3. Wang, M., Herrmann, C. J., Simonovic, M., Szklarczyk, D. & von Mering, C. Version 4.0 of PaxDb: Protein abundance data, integrated across model organisms, tissues, and cell-lines. *Proteomics* **15**, 3163–3168 (2015).
4. Johnson, P. R., Swanson, R., Rakhilina, L. & Hochstrasser, M. Degradation signal masking by heterodimerization of MATα2 and MATa1 blocks their mutual destruction by the ubiquitin-proteasome pathway. *Cell* **94**, 217–227 (1998).
5. Laney, J. D., Mobley, E. F. & Hochstrasser, M. The Short-Lived Matα2 Transcriptional Repressor Is Protected from Degradation In Vivo by Interactions with Its Corepressors Tup1 and Ssn6. *Mol. Cell. Biol.* **26**, 371–380 (2006).
6. Hickey, C. M. & Hochstrasser, M. STUbL-mediated degradation of the transcription factor MATα2 requires degradation elements that coincide with corepressor binding sites. *Mol. Biol. Cell* **26**, 3401–3412 (2015).
7. Giubettini, M. *et al.* Control of Aurora-A stability through interaction with TPX2. *J. Cell Sci.* **124**, 113–122 (2011).
8. Song, L. & Rape, M. Regulated Degradation of Spindle Assembly Factors by the Anaphase-Promoting Complex. *Mol. Cell* **38**, 369–382 (2010).
9. von Klitzing, C. *et al.* APC/C Cdh1-mediated degradation of the F-box protein NIPA is regulated by its association with Skp1. *PLoS One* **6**, e28998 (2011).
10. Wu, W. *et al.* HERC2 is an E3 ligase that targets BRCA1 for degradation. *Cancer Res.* **70**, 6384–6392 (2010).
11. Rezvani, K. *et al.* UBXD4, a UBX-containing protein, regulates the cell surface number and stability of α3-containing nicotinic acetylcholine receptors. *J. Neurosci.* **29**, 6883–6896 (2009).
12. Bedford, F. K. *et al.* GABAA receptor cell surface number and subunit stability are regulated by the ubiquitin-like protein Plic-1. *Nat. Neurosci.* **4**, 908–916 (2001).
13. Saliba, R. S., Pangalos, M. & Moss, S. J. The ubiquitin-like protein plic-1 enhances the membrane insertion of GABAA receptors by increasing their stability within the endoplasmic reticulum. *J. Biol. Chem.* **283**, 18538–18544 (2008).
14. Ratnam, J. & Teichberg, V. I. Neurofilament-light increases the cell surface expression of the N-methyl-D-aspartate receptor and prevents its ubiquitination. *J. Neurochem.* **92**, 878–885 (2005).
15. Yu, C., Cresswell, J., Löffler, M. G. & Bogan, J. S. The Glucose transporter 4-regulating protein TUG is essential for highly insulin-responsive glucose uptake in 3T3-L1 adipocytes. *J. Biol. Chem.* **282**, 7710–7722 (2007).
16. Soloviev, M. M. Detection of a surface-exposed PEST like sequence in the metabotropic glutamate receptor mGluR1α. *Bioinformatics* **16**, 837–838 (2000).
17. Bonifacino, J. S., Cosson, P. & Klausner, R. D. Colocalized transmembrane determinants for ER degradation and subunit assembly explain the intracellular fate of TCR chains. *Cell* **63**, 503–513 (1990).
18. Kopski, K. M. & Huffaker, T. C. Suppressors of the ndc10-2 mutation: A role for the

ubiquitin system in Saccharomyces cerevisiae kinetochore function. *Genetics* **147**, 409–420 (1997).
19. Evans, E. L. *et al.* Dimer formation and conformational flexibility ensure cytoplasmic stability and nuclear accumulation of Elk-1. *Nucleic Acids Res.* **39**, 6390–6402 (2011).
20. Zhou, S. & DeWille, J. W. Proteasome-mediated CCAAT/enhancer-binding protein δ (C/EBPδ) degradation is ubiquitin-independent. *Biochem. J.* **405**, 341–349 (2007).
21. Carrero, Z. I., Kollareddy, M., Chauhan, K. M., Ramakrishnan, G. & Martinez, L. A. Mutant p53 protects ETS2 from non-canonical COP1/DET1 dependent degradation. *Oncotarget* **7**, 12554–12567 (2016).
22. Yoshida, M. *et al.* Poly(A) binding protein (PABP) homeostasis is mediated by the stability of its inhibitor, Paip2. *EMBO J.* **25**, 1934–1944 (2006).
23. Campanero, M. R. & Flemington, E. K. Regulation of E2F through ubiquitin-proteasome-dependent degradation: Stabilization by the pRB tumor suppressor protein. *Proc. Natl. Acad. Sci. U. S. A.* **94**, 2221–2226 (1997).
24. Hofmann, F., Martelli, F., Livingston, D. M. & Wang, Z. The retinoblastoma gene product protects E2F-1 from degradation by the ubiquitin-proteasome pathway. *Genes Dev.* **10**, 2949–2959 (1996).
25. Hateboer, G., Kerkhoven, R. M., Shvarts, A., Bernards, R. & Beijersbergen, R. L. Degradation of E2F by the ubiquitin-proteasome pathway: Regulation by retinoblastoma family proteins and adenovirus transforming proteins. *Genes Dev.* **10**, 2960–2970 (1996).
26. Miyake, S. *et al.* Cells Degrade a Novel Inhibitor of Differentiation with E1A-Like Properties upon Exiting the Cell Cycle. *Mol. Cell. Biol.* **20**, 8889–8902 (2000).
27. Bush, J. R. & Wevrick, R. The Prader-Willi syndrome protein necdin interacts with the E1A-like inhibitor of differentiation EID-1 and promotes myoblast differentiation. *Differentiation* **76**, 994–1005 (2008).
28. Zhang, C. *et al.* Peptidic degron in EID1 is recognized by an SCF E3 ligase complex containing the orphan F-box protein FBXO21. *Proc. Natl. Acad. Sci. U. S. A.* **112**, 15372–15377 (2015).
29. Mathes, E., O'Dea, E. L., Hoffmann, A. & Ghosh, G. NF-κB dictates the degradation pathway of IκBα. *EMBO J.* **27**, 1357–1367 (2008).
30. Fortmann, K. T., Lewis, R. D., Ngo, K. A., Fagerlund, R. & Hoffmann, A. A Regulated, Ubiquitin-Independent Degron in IκBα. *J. Mol. Biol.* **427**, 2748–2756 (2015).
31. Mbom, B. C., Siemers, K. A., Ostrowski, M. A., Nelson, W. J. & Barth, A. I. M. Nek2 phosphorylates and stabilizes ß-catenin at mitotic centrosomes downstream of Plk1. *Mol. Biol. Cell* **25**, 977–991 (2014).
32. Shemorry, A., Hwang, C. S. & Varshavsky, A. Control of Protein Quality and Stoichiometries by N-Terminal Acetylation and the N-End Rule Pathway. *Mol. Cell* **50**, 540–551 (2013).
33. Keppler, B. R. & Archer, T. K. Ubiquitin-dependent and ubiquitin-independent control of subunit stoichiometry in the SWI/SNF complex. *J. Biol. Chem.* **285**, 35665–35674 (2010).
34. Sohn, D. H. *et al.* SRG3 interacts directly with the major components of the SWI/SNF chromatin remodeling complex and protects them from proteasomal degradation. *J. Biol. Chem.* **282**, 10614–10624 (2007).
35. Lécuyer, E. *et al.* Protein stability and transcription factor complex assembly determined by the SCL-LMO2 interaction. *J. Biol. Chem.* **282**, 33649–33658 (2007).

36. Xu, Z. *et al.* Single-stranded DNA-binding proteins regulate the abundance of LIM domain and LIM domain-binding proteins. *Genes Dev.* **21**, 942–955 (2007).
37. Ostendorff, H. P. *et al.* Ubiquitination-dependent cofactor exchange on LIM homeodomain transcription factors. *Nature* **416**, 99–103 (2002).
38. Güngör, C. *et al.* Proteasomal selection of multiprotein complexes recruited by LIM homeodomain transcription factors. *Proc. Natl. Acad. Sci. U. S. A.* **104**, 15000–15005 (2007).
39. Hiratani, I., Yamamoto, N., Mochizuki, T., Ohmori, S. Y. & Taira, M. Selective degradation of excess Ldb1 by Rnf12/RLIM confers proper Ldb1 expression levels and Xlim-1/Ldb1 stoichiometry in Xenopus organizer functions. *Development* **130**, 4161–4175 (2003).
40. Weihe, U., Milán, M. & Cohen, S. M. Regulation of Apterous activity in Drosophila wing development. *Development* **128**, 4615–4622 (2001).
41. Buschmann, T. *et al.* Stabilization and Activation of p53 by the Coactivator Protein TAF II31. *J. Biol. Chem.* **276**, 13852–13857 (2001).
42. Maheswaran, S., Englert, C., Bennett, P., Heinrich, G. & Haber, D. A. The WT1 gene product stabilizes p53 and inhibits p53-mediated apoptosis. *Genes Dev.* **9**, 2143–2156 (1995).
43. An, W. G. *et al.* Stabilization of wild-type p53 by hypoxia-inducible factor 1α. *Nature* **392**, 406–508 (1998).
44. Yuan, Z. M. *et al.* Role for p300 in stabilization of p53 in the response to DNA damage. *J. Biol. Chem.* **274**, 1883–1886 (1999).
45. Kawai, H., Nie, L., Wiederschain, D. & Yuan, Z. M. Dual Role of p300 in the Regulation of p53 Stability. *J. Biol. Chem.* **276**, 45928–45932 (2001).
46. Park, Y., Yoon, S. K. & Yoon, J. B. TRIP12 functions as an E3 ubiquitin ligase of APP-BP1. *Biochem. Biophys. Res. Commun.* **374**, 294–298 (2008).
47. Kuo, M. L., Den Besten, W., Bertwistle, D., Roussel, M. F. & Sherr, C. J. N-terminal polyubiquitination and degradation of the Arf tumor suppressor. *Genes Dev.* **18**, 1862–1874 (2004).
48. Abu-Shaar, M. & Mann, R. S. Generation of multiple antagonistic domains along the proximodistal axis during Drosophila leg development. *Development* **125**, 3821–3830 (1998).
49. Otto, T. *et al.* Stabilization of N-Myc Is a Critical Function of Aurora A in Human Neuroblastoma. *Cancer Cell* **15**, 67–78 (2009).
50. Wang, N., Guo, L., Rueda, B. R. & Tilly, J. L. Cables1 protects p63 from proteasomal degradation to ensure deletion of cells after genotoxic stress. *EMBO Rep.* **11**, 633–639 (2010).
51. Kim, T. S. *et al.* Interaction of Hsp90 with ribosomal proteins protects from ubiquitination and proteasome-dependent degradation. *Mol. Biol. Cell* **17**, 824–833 (2006).
52. Gao, L. & Harhaj, E. W. HSP90 Protects the Human T-Cell Leukemia Virus Type 1 (HTLV-1) Tax Oncoprotein from Proteasomal Degradation To Support NF- B Activation and HTLV-1 Replication. *J. Virol.* **87**, 13640–13654 (2013).
53. Zheng, Y., Ao, Z., Wang, B., Jayappa, K. D. & Yao, X. Host protein Ku70 binds and protects HIV-1 integrase from proteasomal degradation and is required for HIV replication. *J. Biol. Chem.* **286**, 17722–17735 (2011).
54. Llano, M., Delgado, S., Vanegas, M. & Poeschla, E. M. Lens epithelium-derived growth

factor/p75 prevents proteasomal degradation of HIV-1 integrase. *J. Biol. Chem.* **279**, 55570–55577 (2004).

55. Baylot, V. *et al.* Targeting TCTP as a new therapeutic strategy in castration-resistant prostate cancer. *Mol. Ther.* **20**, 2244–2256 (2012).
56. Asher, G., Tsvetkov, P., Kahana, C. & Shaul, Y. A mechanism of ubiquitin-independent proteasomal degradation of the tumor suppressors p53 and p73. *Genes Dev.* **19**, 316–321 (2005).
57. Amir, S., Wang, R., Matzkin, H., Simons, J. W. & Mabjeesh, N. J. MSF-A interacts with hypoxia-lnducible factor-1αand augments hypoxia-inducible factor transcriptional activation to affect tumorigenicity and angiogenesis. *Cancer Res.* **66**, 856–866 (2006).
58. Marcus, E. A. *et al.* Septin oligomerization regulates persistent expression of ErbB2/HER2 in gastric cancer cells. *Biochem. J.* **473**, 1703–1718 (2016).
59. Yang, J. *et al.* HSP70 protects BCL2L12 and BCL2L12A from N-terminal ubiquitination-mediated proteasomal degradation. *FEBS Lett.* **583**, 1409–1414 (2009).
60. Kang, C. M., Kang, S., Park, Y. S. & Yun, C. W. Physical interaction between Sit1 and Aft1 upregulates FOB uptake activity by inhibiting protein degradation of Sit1 in Saccharomyces cerevisiae. *FEMS Yeast Res.* **15**, fov080 (2015).
61. Khanna, R., Lee, E. J. & Papazian, D. M. Transient calnexin interaction confers long-term stability on folded K+ channel protein in the ER. *J. Cell Sci.* **117**, 2897–2908 (2004).
62. Draheim, K. M. *et al.* CCM2-CCM3 interaction stabilizes their protein expression and permits endothelial network formation. *J. Cell Biol.* **208**, 987–1001 (2015).
63. Dimitrova, Y. N. *et al.* Direct ubiquitination of β-catenin by Siah-1 and regulation by the exchange factor TBL1. *J. Biol. Chem.* **285**, 13507–13516 (2010).
64. Huang, J. *et al.* Exchange factor TBL1 and arginine methyltransferase PRMT6 cooperate in protecting g protein pathway suppressor 2 (GPS2) from proteasomal degradation. *J. Biol. Chem.* **290**, 19044–19054 (2015).
65. Crystal, A. S. *et al.* Presenilin Modulates Pen-2 Levels Posttranslationally by Protecting It from Proteasomal Degradation. *Biochemistry* **43**, 3555–3563 (2004).
66. Kahana, C., Asher, G. & Shaul, Y. Mechanisms of protein degradation: An odyssey with ODC. *Cell Cycle* **4**, 1461–1464 (2005).
67. Muromoto, R. *et al.* Physical and Functional Interactions between Daxx and DNA Methyltransferase 1-Associated Protein, DMAP1. *J. Immunol.* **172**, 2985–2993 (2004).
68. Xie, Y., Burcu, M., Linn, D. E., Qiu, Y. & Baer, M. R. Pim-1 kinase protects P-glycoprotein from degradation and enables its glycosylation and cell surface expression. *Mol. Pharmacol.* **78**, 310–318 (2010).
69. Wang, W.-T., Tsai, T.-Y., Chao, C.-H., Lai, B.-Y. & Wu Lee, Y.-H. Y-Box Binding Protein 1 Stabilizes Hepatitis C Virus NS5A via Phosphorylation-Mediated Interaction with NS5A To Regulate Viral Propagation. *J. Virol.* **89**, 11584–11602 (2015).
70. Fearns, C., Pan, Q., Mathison, J. C. & Chuang, T. H. Triad3A regulates ubiquitination and proteasomal degradation of RIP1 following disruption of Hsp90 binding. *J. Biol. Chem.* **281**, 34592–34600 (2006).
71. Lewis, J. *et al.* Disruption of Hsp96 function results in degradation of the death domain kinase, receptor-interacting protein (RIP), and blockage of tumor necrosis factor-induced nuclear factor-κB activation. *J. Biol. Chem.* **275**, 10519–10526 (2000).
72. Teng, Y., Ngoka, L., Mei, Y., Lesoon, L. & Cowell, J. K. HSP90 and HSP70 Proteins are essential for stabilization and activation of WASF3 metastasis-promoting protein. *J.*

Biol. Chem. **287**, 10051–10059 (2012).
73. Park, S. J., Suetsugu, S. & Takenawa, T. Interaction of HSP90 to N-WASP leads to activation and protection from proteasome-dependent degradation. *EMBO J.* **24**, 1557–1570 (2005).
74. Cheung, L. W. T. *et al.* Regulation of the PI3K pathway through a p85α monomer–homodimer equilibrium. *Elife* **4**, (2015).
75. Liu, M., Hsu, J., Chan, C., Li, Z. & Zhou, Q. The Ubiquitin Ligase Siah1 Controls ELL2 Stability and Formation of Super Elongation Complexes to Modulate Gene Transcription. *Mol. Cell* **46**, 325–334 (2012).
76. Yang, W., Dicker, D. T., Chen, J. & El-Deiry, W. S. CARPs enhance p53 turnover by degrading 14-3-3σ and stabilizing MDM2. *Cell Cycle* **7**, 670–682 (2008).
77. Gong, Y. *et al.* Sterol-regulated ubiquitination and degradation of Insig-1 creates a convergent mechanism for feedback control of cholesterol synthesis and uptake. *Cell Metab.* **3**, 15–24 (2006).
78. Csomos, R. A., Brady, G. F. & Duckett, C. S. Enhanced cytoprotective effects of the inhibitor of apoptosis protein cellular IAP1 through stabilization with TRAF2. *J. Biol. Chem.* **284**, 20531–20539 (2009).
79. Tomlinson, V. *et al.* JNK phosphorylates Yes-associated protein (YAP) to regulate apoptosis. *Cell Death Dis.* **1**, e29–e29 (2010).
80. Levy, D., Adamovich, Y., Reuven, N. & Shaul, Y. The Yes-associated protein 1 stabilizes p73 by preventing Itch-mediated ubiquitination of p73. *Cell Death Differ.* **14**, 743–751 (2007).
81. Fang, Q. *et al.* HSP90 regulates DNA repair via the interaction between XRCC1 and DNA polymerase β. *Nat. Commun.* **5**, 5513 (2014).
82. Oda, T., Hayano, T., Miyaso, H., Takahashi, N. & Yamashita, T. Hsp90 regulates the Fanconi anemia DNA damage response pathway. *Blood* **109**, 5016–5026 (2007).
83. Ellison, M. A., Gearheart, C. M., Porter, C. C. & Ambruso, D. R. IFN-γ alters the expression of diverse immunity related genes in a cell culture model designed to represent maturing neutrophils. *PLoS One* **12**, e0185956 (2017).
84. Wang, W., Ungermannova, D., Jin, J., Harper, J. W. & Liu, X. Negative regulation of SCFSkp2 ubiquitin ligase by TGF-β signaling. *Oncogene* **23**, 1064–1075 (2004).
85. Chou, D. M. & Elledge, S. J. Tipin and Timeless form a mutually protective complex required for genotoxic stress resistance and checkpoint function. *Proc. Natl. Acad. Sci. U. S. A.* **103**, 18143–18147 (2006).
86. Ji, L. *et al.* The SIAH E3 ubiquitin ligases promote Wnt/β-catenin signaling through mediating Wnt-induced Axin degradation. *Genes Dev.* **31**, 904–915 (2017).
87. Ricke, R. M., Jeganathan, K. B. & van Deursen, J. M. Bub1 overexpression induces aneuploidy and tumor formation through Aurora B kinase hyperactivation. *J. Cell Biol.* **193**, 1049–1064 (2011).
88. Baker, D. J., Jin, F., Jeganathan, K. B. & van Deursen, J. M. Whole Chromosome Instability Caused by Bub1 Insufficiency Drives Tumorigenesis through Tumor Suppressor Gene Loss of Heterozygosity. *Cancer Cell* **16**, 475–486 (2009).
89. Bianco, J. N. *et al.* Overexpression of Claspin and Timeless protects cancer cells from replication stress in a checkpoint-independent manner. *Nat. Commun.* **10**, 910 (2019).
90. Tsimaratou, K. *et al.* Evaluation of claspin as a proliferation marker in human cancer and normal tissues. *J. Pathol.* **211**, 331–339 (2007).
91. Schaub, F. X. *et al.* Pan-cancer Alterations of the MYC Oncogene and Its Proximal

Network across the Cancer Genome Atlas. *Cell Syst.* **6**, 282-300.e2 (2018).
92. Bykov, V. J. N., Eriksson, S. E., Bianchi, J. & Wiman, K. G. Targeting mutant p53 for efficient cancer therapy. *Nat. Rev. Cancer* **18**, 89–102 (2018).
93. Brosh, R. & Rotter, V. When mutants gain new powers: News from the mutant p53 field. *Nat. Rev. Cancer* **9**, 701–713 (2009).
94. Zhang, W. *et al.* Skp2 is over-expressed in breast cancer and promotes breast cancer cell proliferation. *Cell Cycle* **15**, 1344–1351 (2016).
95. Zhong, K., Yang, F., Han, Q., Chen, J. & Wang, J. Skp2 expression has different clinicopathological and prognostic implications in lung adenocarcinoma and squamous cell carcinoma. *Oncol. Lett.* **16**, 2873–2880 (2018).
96. Pan, H. W. *et al.* Role of L2DTL, cell cycle-regulated nuclear and centrosome protein, in aggressive hepatocellular carcinoma. *Cell Cycle* **5**, 2676–2687 (2006).
97. Ueki, T. *et al.* Involvement of elevated expression of multiple cell-cycle regulator, DTL/RAMP (denticleless/RA-regulated nuclear matrix associated protein), in the growth of breast cancer cells. *Oncogene* **27**, 5672–5683 (2008).
98. Kobayashi, H. *et al.* Overexpression of denticleless E3 ubiquitin protein ligase homolog (DTL) is related to poor outcome in gastric carcinoma. *Oncotarget* **6**, 36615–36624 (2015).
99. Kudo, Y. *et al.* Role of F-Box Protein βTrcp1 in Mammary Gland Development and Tumorigenesis. *Mol. Cell. Biol.* **24**, 8184–8194 (2004).
