# Supplementary material for: Degron masking outlines degronons, co-degrading functional modules in the proteome
Source: Commun Biol. 2022 May 11;5:445. doi: 10.1038/s42003-022-03391-z (PMC9095673; doi:10.1038/s42003-022-03391-z)
Supplement: Supplementary file 3 — Description of Additional Supplementary Files [file 42003_2022_3391_MOESM3_ESM.pdf]

Description of Additional Supplementary Files

File name: Supplementary Data 1:
Description: Annotated degrons and degradation substrates. List of degradation substrates with information about their experimentally annotated degrons.

File name: Supplementary Data 2:
Description: UPS related GO terms. List of Gene Ontology terms linked to components of the Ubiquitin-proteasome system.

File name: Supplementary Data 3:
Description: Primary degron overlapping features. UniProt annotations and descriptions of protein functional features (binding sites, functional sites, etc.) that overlap with primary degrons.

File name: Supplementary Data 4:
Description: Secondary degron overlapping features. UniProt annotations and descriptions of protein functional features (binding sites, functional sites, etc.) that overlap with secondary degrons.

File name: Supplementary Data 5:
Description: Tertiary degron overlapping features. UniProt annotations and descriptions of protein functional features (binding sites, functional sites, etc.) that overlap with tertiary degrons.

File name: Supplementary Data 6:
Description: Primary degron overlapping motifs. Details of overlapping and adjacent ELMs relative to primary degrons.

File name: Supplementary Data 7:
Description: Secondary degron overlapping motifs. Details of overlapping and adjacent ELMs relative to secondary degrons.

File name: Supplementary Data 8:
Description: Tertiary degron overlapping motifs. Details of overlapping and adjacent ELMs relative to tertiary degrons.

File name: Supplementary Data 9:
Description: Kd dataset of competing interactions. Experimentally measured dissociation constants for substrate-E3 and substrate-alternate partner (AP) interactions.

File name: Supplementary Data 10:
Description: Protein abundance dataset details. Human protein abundance datasets obtained from PaxDb and their grouping as used in this study.

File name: Supplementary Data 11:
Description: Functionally connected pathways delineated from PPI network. List of high Gene Ontology Biological Process semantic similarity pathways identified from the Collins PPI
